# Supplementary material for: Temporal Analysis Reveals the Transient Differential Expression of Transcription Factors That Underlie the Trans-Differentiation of Human Monocytes to Macrophages
Source: Int J Mol Sci. 2022 Dec 13;23(24):15830. doi: 10.3390/ijms232415830 (PMC9781183; doi:10.3390/ijms232415830)
Supplement: Supplementary file 1 [file ijms-23-15830-s001.zip › ijms-1968353-supplementary.pdf]

Supplementary materials

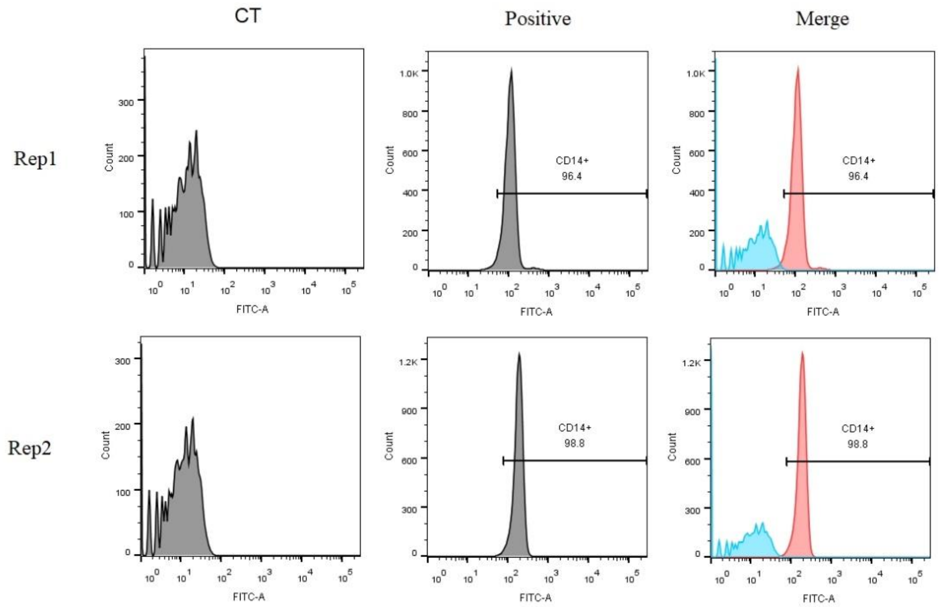

Figure S1. Flow cytometric analysis (FACS) of purified primary monocytes based on the monocyte cell marker, CD14. The percentage of $CD14^{+}$ cells are shown in the insets.

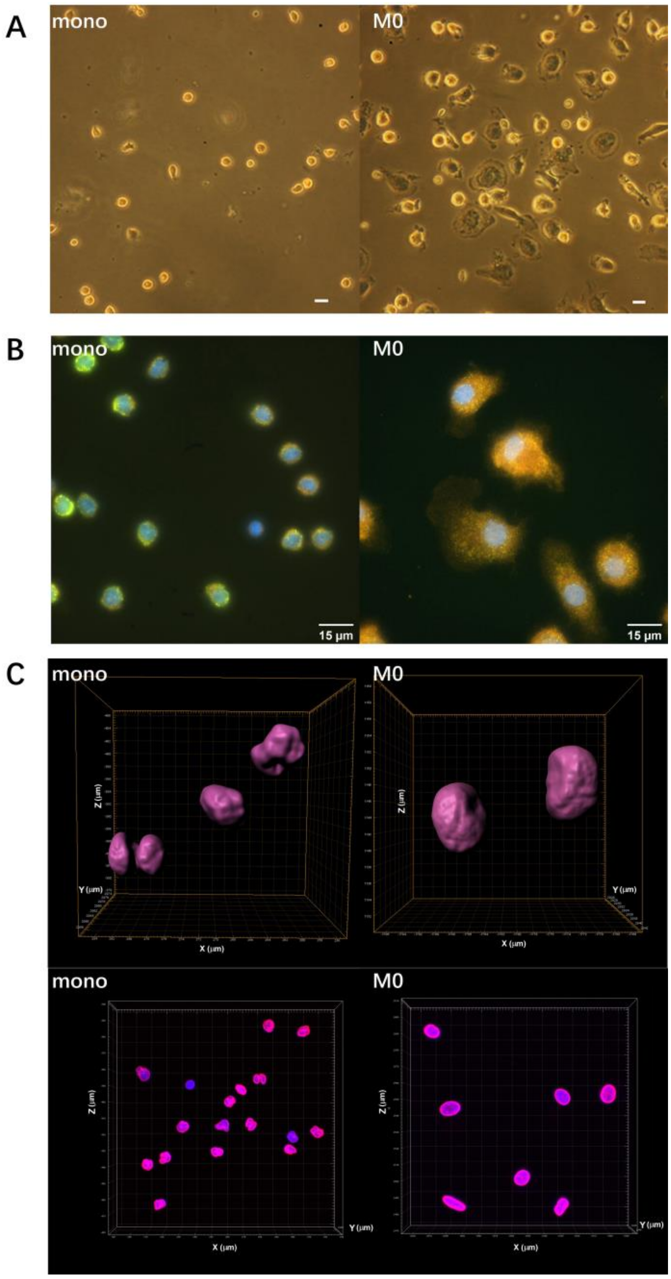

Figure S2. Morphological and immunofluorescence characterization of monocytes and trans-differentiated macrophages. (A) Cell morphology under phase contrast microscopy (40x). Shown are the purified monocytes (left) and the cells 7-days following NHS addition (M0 macrophages). Scale bar = 15 µm (B) Immunofluorescence of cells labeled with the monocyte cell marker CD14 (green), M0 macrophage marker CD68 (orange) or DAPI for the nuclei (blue). Scale bar = 15 µm (C) Immunofluorescence of cells labeled with the nuclear membrane protein Lamin B to show nuclear morphology. The upper panel is a higher magnified view to better show the change in nuclear shape.

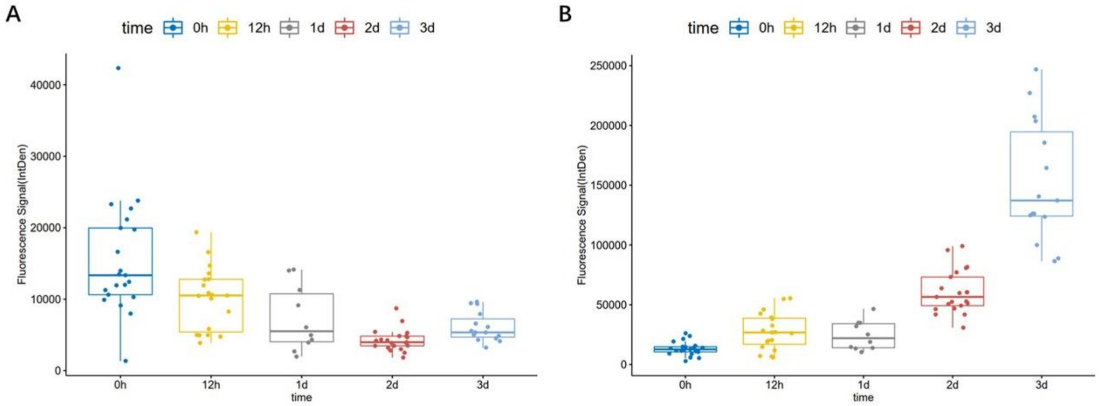

Figure S3. Immunofluorescence signal of CD68 (A) and CD14 (B) of cells during the trans-differentiation process.

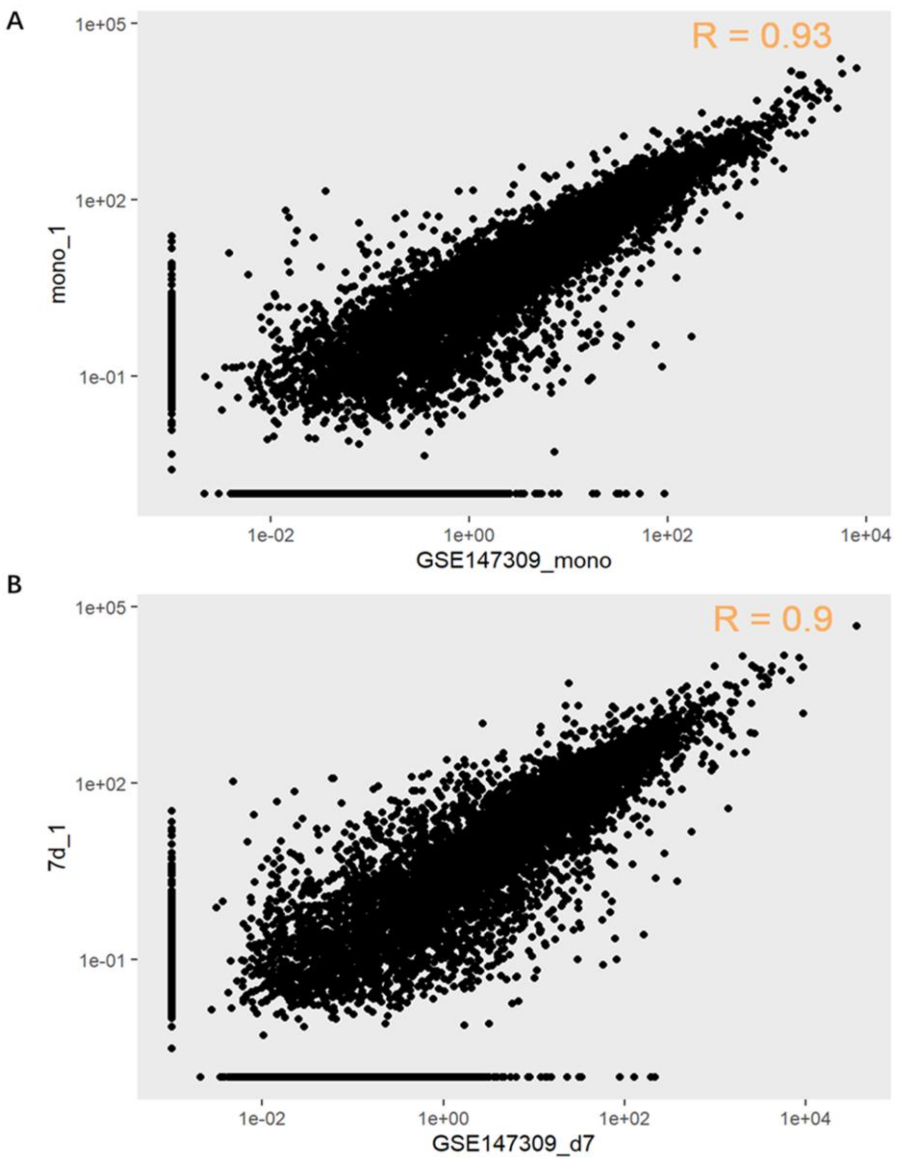

Figure S4. Comparison of the gene expression data between our results and those published previously[32]. (A) Comparison between our monocytes RNA-seq data (mono_1) with the published data (Spearman R=0.93). (B) Comparison between our M0 macrophage sample (7 d_1) with the published data (Spearman' R=0.90).

A

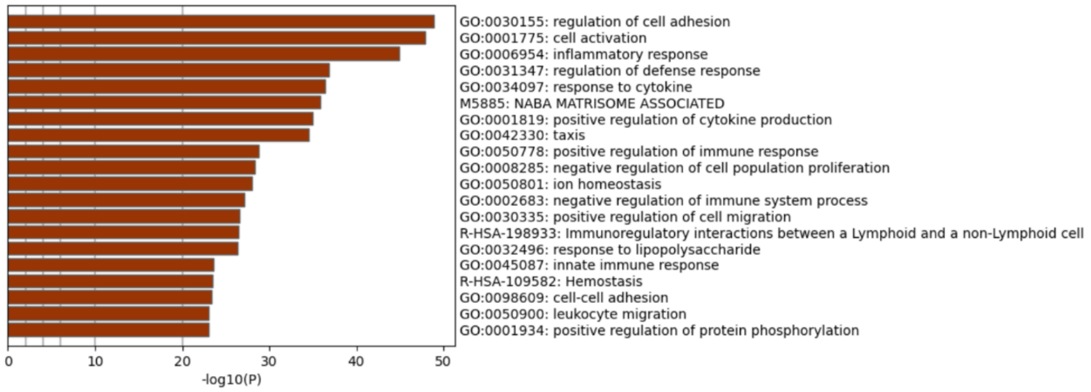

B

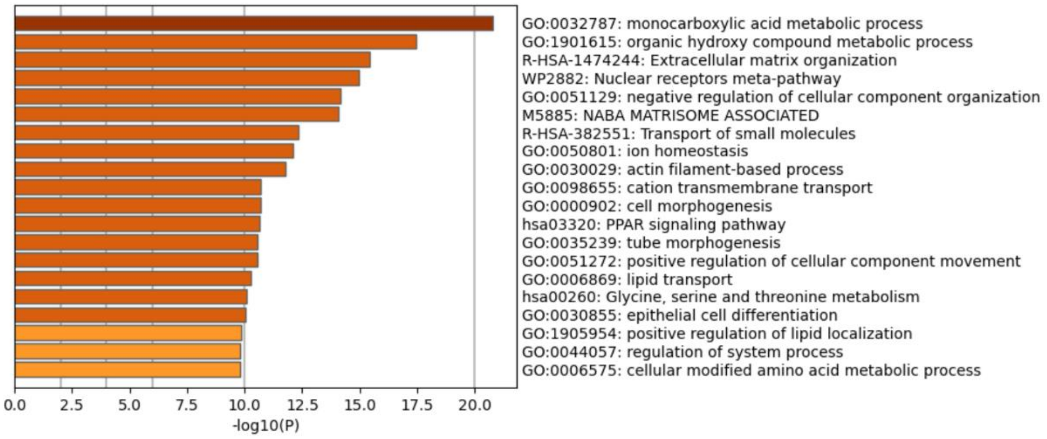

C

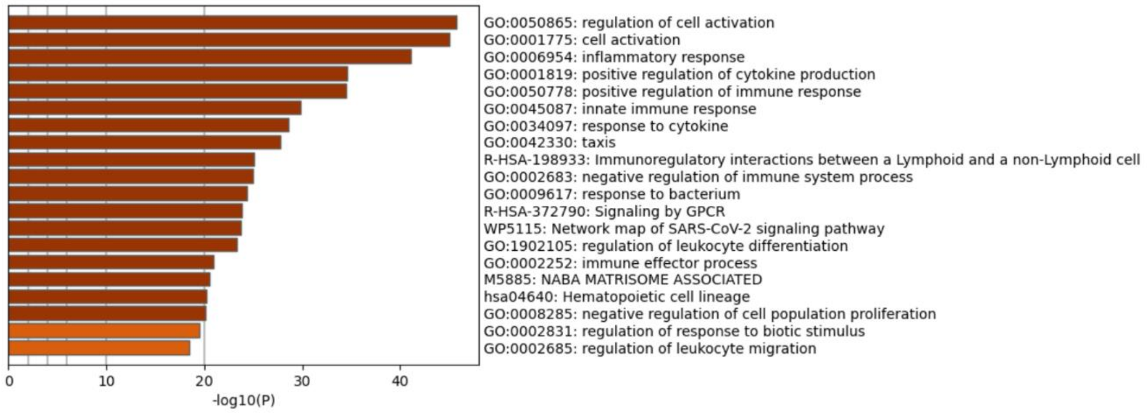

Figure S5. GO analysis of enriched annotated features in all DEGs (A), the up-regulated DEGs (B), and the down-regulated DEGs (C).

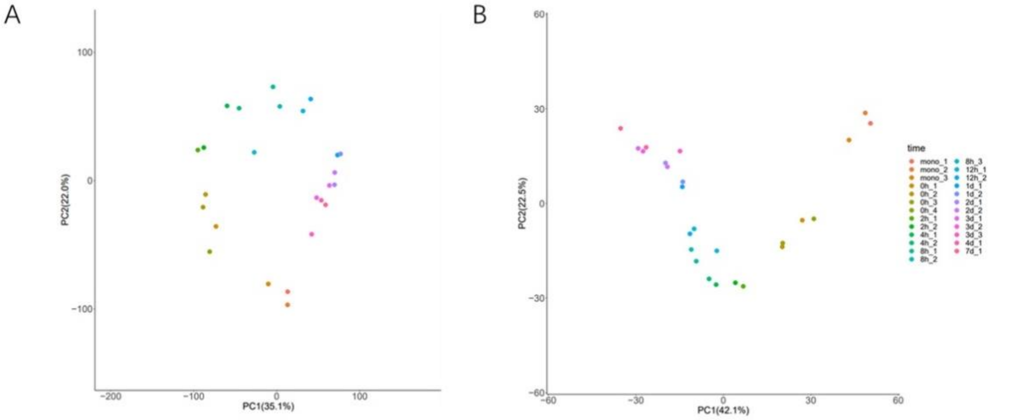

Figure S6. Temporal changes in the transcriptome during the trans-differentiation of monocytes to M0 macrophages of all replicates. (A) PCA analysis of the whole transcriptome. (B) PCA analysis of highly-expressed DEGs.

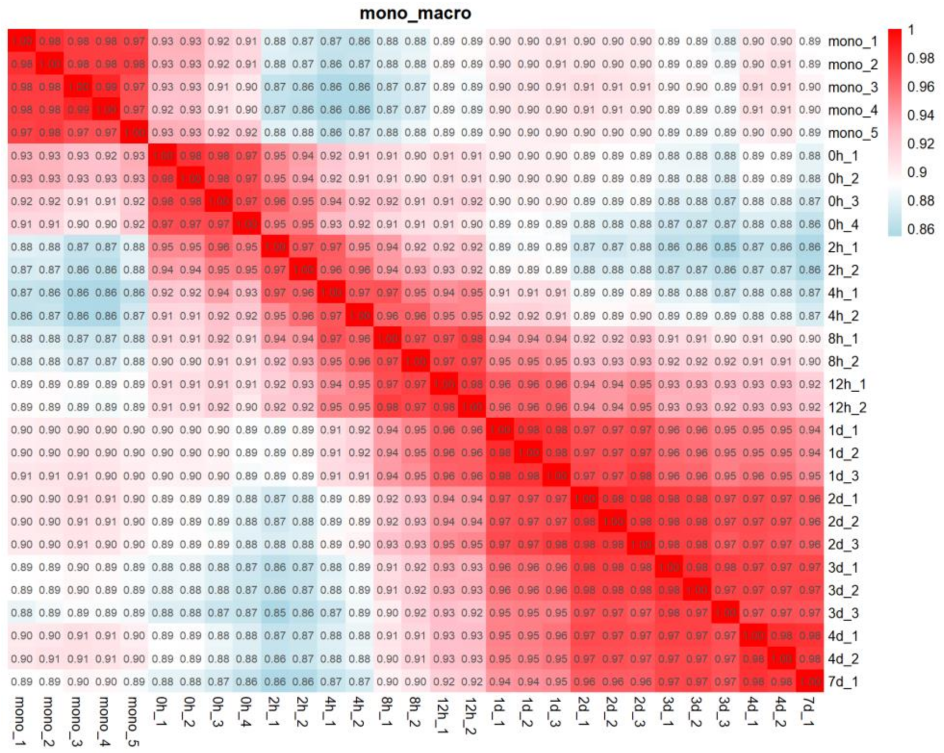

Figure S7. Extent of correlation between all RNA-seq data samples. Shown are the pairwise Spearman correlation coefficients.

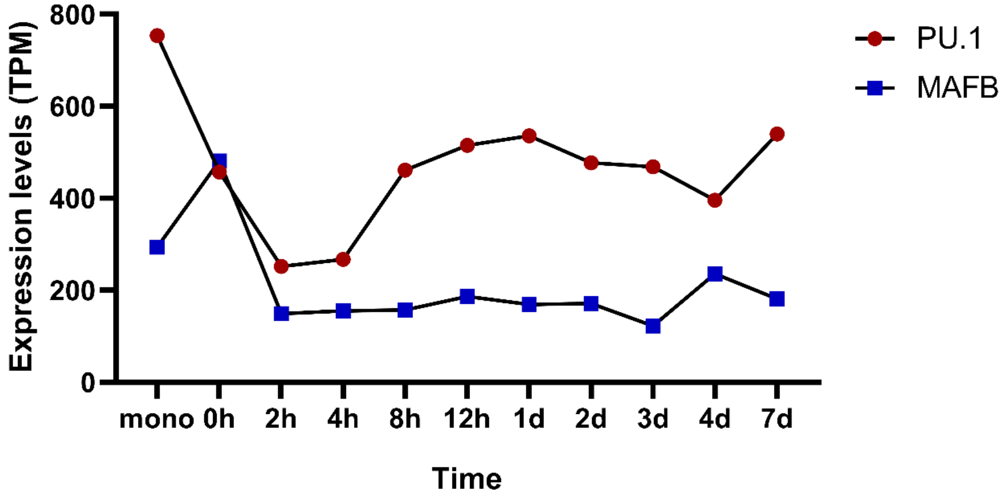

Figure S8. The expression levels of the well-known TFs, MAFB and PU.1, throughout the process.

A

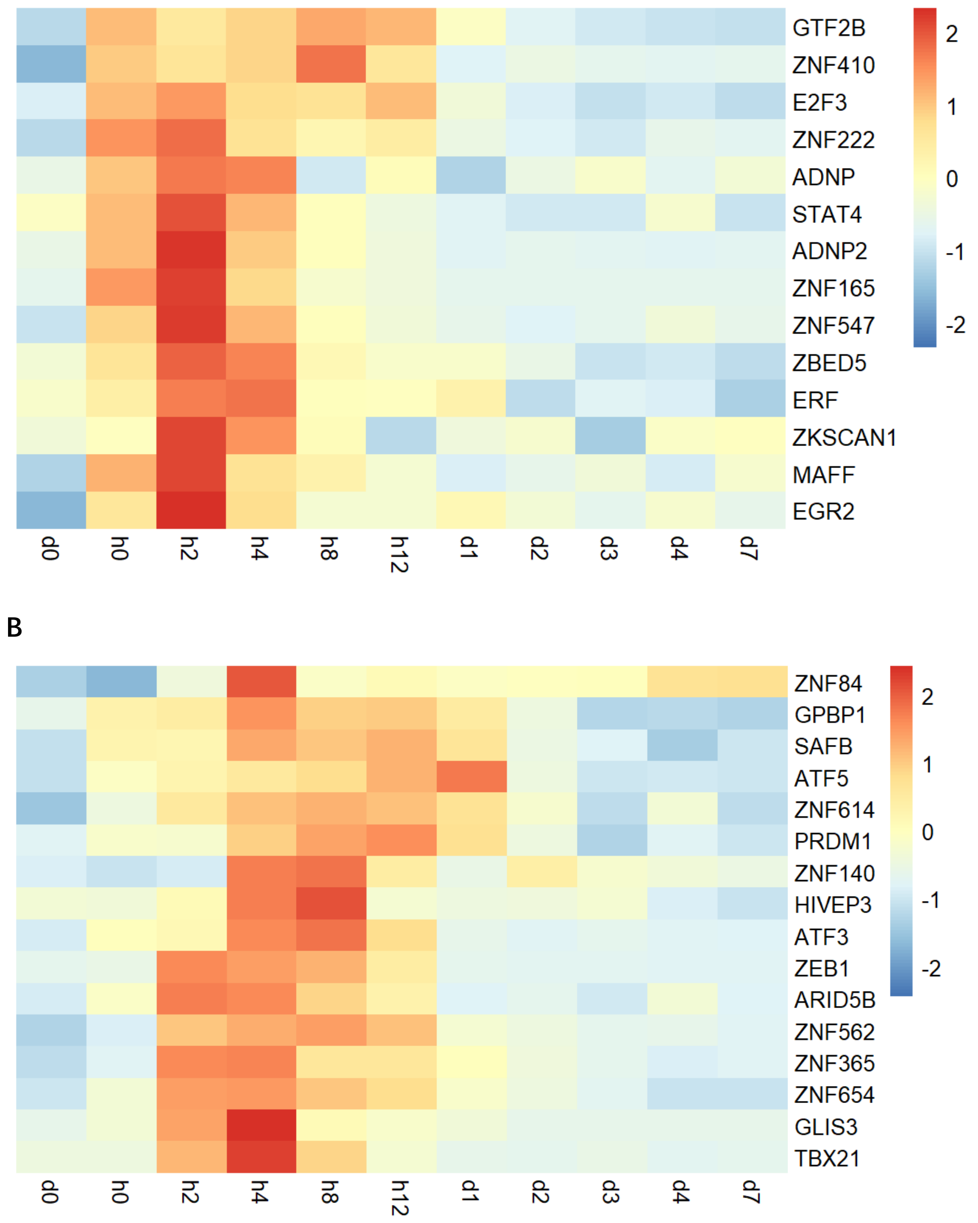

Figure S9. Potential AP-1 target TFs whose expression peaks at later time periods following addition of NHS. Up-regulated group 2 (A) and group 3 (B) genes with AP-1 binding motifs within their promoter regions.

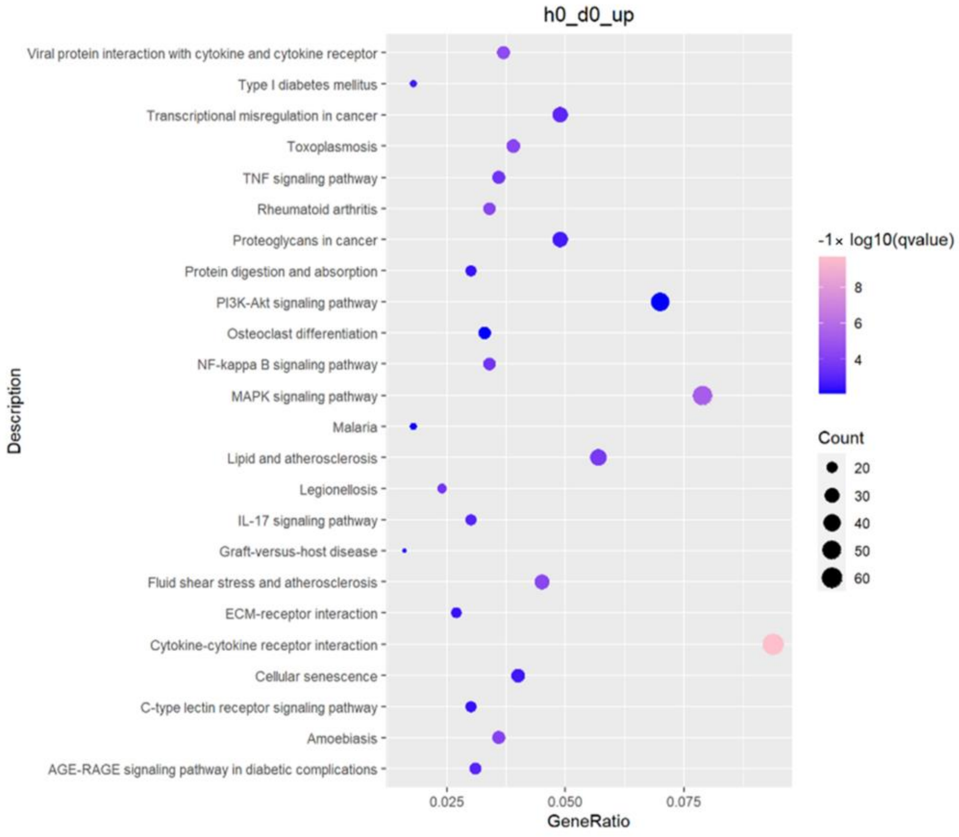

Figure S10. KEGG analysis of up-regulated genes at 0 h compared with purified monocytes. Terms with a p value < 0.01 and *p.* adjust < 0.05 were considered as significantly enriched.
